# Evaluation of Custom Microalgae-Based Bioink Formulations for Optimized Green Bioprinting

**DOI:** 10.3390/ma18040753

**Published:** 2025-02-08

**Authors:** Olubusuyi Ayowole, Justin Lapp, Bashir Khoda

**Affiliations:** Department of Mechanical Engineering, University of Maine, Orono, ME 04469, USA; olubusuyi.ayowole@maine.edu (O.A.); justin.lapp@maine.edu (J.L.)

**Keywords:** bioink, bioprinting, absorbance, microalgae, hydrogel

## Abstract

Green bioprinting, from the context of merging 3D bioprinting with microalgae cell organization, holds promise for industrial-scale optimization. This study employs spectrophotometric analysis to explore post-bioprinting cell growth density variation within hybrid hydrogel biomaterial scaffolds. Three hydrogel biomaterials—Alginic acid sodium salt (ALGINATE), Nanofibrillated Cellulose (NFC)—TEMPO, and CarboxyMethyl Cellulose (CMC)—are chosen for their scaffolding capabilities. Bioink development and analysis of their impact on cell proliferation and morphology are conducted. Chlorella microalgae cell growth within hydrogel compositions is probed using absorbance measurements, with additional assessment of shear thinning properties. Notably, NFC exhibits reduced shear thinning compared to CMC. Results reveal that while mono-hydrogel substrates with pronounced adhesion inhibit Chlorella cell proliferation, alginate fosters increased cell concentration alongside a slight viscosity rise.

## 1. Introduction

Green bioprinting, the use of 3D printing with scaffolded plant cells, has a wide range of potential applications, from industrial-scale bioplastic production to sustainable biofuels. Various engineering disciplines have investigated 3D bioprinting through the printing of microalgae [1,2]. This method has the potential to enable the production of artificial leaves, photosynthetic skins, and bio-garments, offering sustainable alternatives in architecture and fashion [3]. Green bioprinting may facilitate the development of nutrient-rich food supplements and advanced drug delivery systems, contributing significantly to healthcare advancements [4]. In environmental engineering, microalgae printing presents solutions for wastewater treatment and carbon capture, thus addressing pollution and climate change challenges [5]. Additionally, the application of green bioprinting extends to biosensors for environmental monitoring and diagnostics [6]. The procedures involved in 3D bioprinting of microalgae offer insights that can advance research towards 3D bioprinting of human tissues and organs, addressing the challenge of organ transplant shortages.

Real-world demonstrations of microalgae bioprinting include the mini t-shirt printed by researchers at the University of Rochester, and Chlorella supplementation studied by Tottori University, Japan [7,8]. Although the origin of this study stems from research based on the 3D bioprinting of blueberry cells, microalgae cells were chosen for preliminary investigation due to their high multiplication rates and high availability compared to the complexity of extracting blueberry stem cells for pre-bioprinting culture.

The objectives of this study aim to comprehensively investigate cell functionality within 3D bio-printed constructs of algae cells incorporated into hybrid hydrogel scaffold bio-environments. This investigation was conducted through spectrophotometric analysis, focusing on variations in cell growth density. The study seeks to identify and establish a procedure for formulating optimum hybrid hydrogel compositions that are best suited for promoting cell multiplication either before or after subjecting microalgae cell-laden custom bioinks to the 3D bioprinting process. Central to the research are phenomena such as cell proliferation and morphology of microalgae cells, particularly in correlation to the post-printing effects of nozzle wall pressure on the cell-laden hybrid hydrogel scaffolds. To achieve this, rheological data of the hydrogel materials were obtained using a plate-and-cone rheometer to understand the shear thinning (i.e., a decrease in a material’s viscosity as the rate of deformation increases.) properties of hydrogel formulations in relation to cell growth or damage. Additionally, the study employed a spectrophotometer to periodically record the absorbance of different bioink compositions derived from hybrid hydrogel scaffolds, which is correlated with cell count. Within each substrate, the analysis assesses the survivability and growth conditions of algae cells, providing an evaluation of each hydrogel scaffold for cell cultivation. Combining rheological studies with evaluations of cell viability through spectroscopy or spectrophotometric analysis helps assess the biocompatibility and effectiveness of bioinks, thereby improving the printing process and the long-term stability of cells in bioprinted constructs [9]. Studies on the bioprintability of microalgae may provide foundational insights for developing protocols applicable to other plant cells with unique properties. For instance, extrusion-based bioprinting of photosynthetic microalgae has demonstrated the potential to create 3D hydrogel scaffolds, which are critical for biotechnological and plant-based material applications [10,11]. The incorporation of microalgae into hydrogels offers a promising approach to sustainable bioprinting, addressing key challenges such as cell viability and scaffold integration, essential for successful applications in environmental biotechnology [12].

The bioprinting of *Arabidopsis* cells under specific nutrient and scaffolding conditions has shown that long-term cell viability and division can be achieved, leading to the formation of microcalli. This process, by analogy, is important for understanding cellular regeneration, with bioprinted cells adapting and exhibiting stem cell-like behaviors by expressing regeneration-related genes [13]. These findings support the integration of plant cell systems into biofabrication, opening new pathways to harness plant properties. Research on the bioprintability of microalgae also provides insights that extend to the 3D bioprinting of other chlorophyll-based plant cells. Advancements in algae-laden hydrogel scaffolds have shown improvements in photosynthetic performance and structural stability, which are essential for applications like oxygen production and carbon capture [14]. These studies emphasize not only the potential of microalgae but also the broader vision of leveraging plant cells for future technological and medical applications, such as the use of antioxidant-rich plant cells like blueberries for combating oxidative stress and radiation damage prevention [15]. Researching the post-3D bioprinting behavior of microalgae bio-constructs, specifically in terms of the variation of algae cell density within different hydrogel compositions, is thus considered a logical and highly impactful step forward.

Biofabrication encompasses the automated generation of biologically functional products through the coordinated assembly of living cells, biomaterials, and bioactive molecules via techniques such as bioprinting or bio-assembly [16,17,18]. Various methods are employed in biofabrication, including 3D bioprinting, scaffold-based tissue engineering, cell sheet engineering, decellularization and recellularization, microfluidics and organ-on-a-chip, electrospinning, self-assembly and tissue fusion, and biomimicry and bioinspired design [19,20]. Among these, 3D bioprinting stands out as a potential game-changer, offering an alternative source of addressing the organ shortage crisis by creating fully functional 3D organs and reducing reliance on donations from living or deceased individuals [21,22], although ethical and legal considerations remain significant hurdles [23]. Notably, 3D bioprinting presents novel solutions for orthopedic implantation and artificial prostheses demonstrating its versatility in the field of medical engineering [24,25]. The impact of 3D bioprinting extends beyond medicine into the food sector, where it has been studied for cultured meat production by providing realistic texture, reducing costs, and enhancing ecological sustainability [26,27].

**Figure 1:**
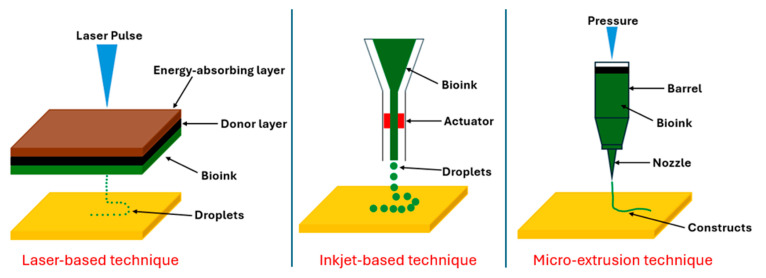
Techniques of 3D bioprinting.

There are three popular 3D bioprinting techniques: inkjet, laser, and micro-extrusion. Inkjet bioprinting offers advantages such as low printing cost, fast printing, and relatively high precision, but it is limited to low-viscosity bioinks and is prone to clogging and drying issues [28]. Laser bioprinting allows high cell densities and resolution and a wide range of bioink viscosity without clogging, but it is relatively time-consuming, costly, and may involve toxic initiators [29]. Micro-extrusion bioprinting, characterized by simple components and control systems, enables printing of heterogeneous constructs and high cell densities, yet it suffers from limited resolution and speed, nozzle clogging, and reduced mammalian cell viability compared to inkjet-based bioprinting [30]. Figure 1 illustrates these common techniques of 3D bioprinting, including laser-based, inkjet-based, and micro-extrusion methods [31]. The micro-extrusion technique is adopted in this study.

## 2. Methodology

### 2.1. Scaffolding Materials

This study explores the impact of different hybrid hydrogel scaffolds on the morphological features of 3D-printed *Chlorella* algae cells. The following bioink scaffolding biomaterials were selected:

- Alginic Acid Sodium Salt (ALGINATE), derived from brown algae, obtained from SIGMA-ALDRICH, Co., St. Louis, MO, USA.
- Nanofibrillated Cellulose (NFC)—TEMPO, acquired from the University of Maine Process Development Center, Orono, ME, USA.
- Medium Viscosity CarboxyMethyl Cellulose (CMC), sourced from SIGMA-ALDRICH, Co., St. Louis, MO, USA.

Alginates are polysaccharide polymers extracted from brown seaweed, providing a conducive microenvironment for cell proliferation when used in bioinks. Alginic acid exhibits shear-thinning behavior, aiding in the extrusion process during 3D bioprinting. It is composed of D-mannuronic acid and L-guluronic acid residues arranged in blocks within the polymer chain [32]. Nanofibrillated Cellulose (NFC), also known as Cellulose Nanofibril (CNF), boasts properties such as high stiffness, strength, and biocompatibility. Extracted from cellulose fibers through mechanical and chemical processes, NFC offers easy film-forming capability [33]. Carboxymethyl Cellulose (CMC) is a water-soluble cellulose derivative prepared by chemical modification. It is characterized by flexibility, stability, and pH sensitivity, making it suitable for various applications, including tissue engineering and 3D bioprinting [34].

The selection of hybrid formulations in this study, by incorporating Alginate, NFC, and CMC, was based on their complementary properties that enable an optimal balance between optical and rheological characteristics. Alginate, with its transparency and biocompatibility, facilitates light transmission, which is crucial for supporting photosynthetic activity. NFC contributes to the structural integrity of the bioink, offering film-forming capabilities without obstructing light penetration. CMC enhances flexibility and stability, further complementing the properties of alginate and NFC.

These hybrid bioinks were designed to maintain transparency for photosynthesis while also providing the necessary rheological properties, particularly viscosity, that support cell proliferation and printability during 3D bioprinting. This approach seeks to lay the groundwork for future research aimed at refining bioink compositions to maximize cell viability, photosynthetic efficiency, and biofabrication success.

**Figure 2:**
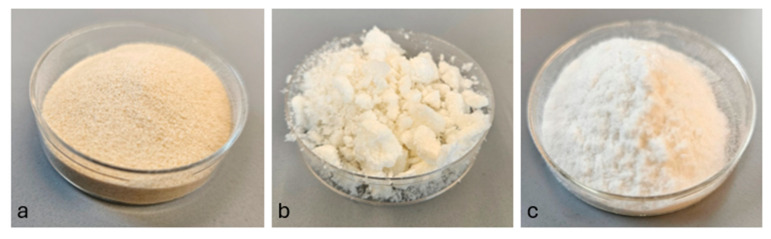
Alginate sample (**a**), NFC sample (**b**), and CMC sample (**c**).

Samples from the laboratory during this study are shown in Figures 2a, 2b and 2c for alginate, NFC, and CMC, respectively.

### 2.2. Preparation of Substrates for Custom Bioinks

An initial set of hydrogel samples was prepared using ALGINATE, NFC, and CMC biomaterials to assess susceptibility to bacterial infection. A 1% (*w*/*v*) solution of each material was dissolved in deionized water, mixed for 12 h, and stored in well plates. The absence of visible black spots indicated the absence of bacterial infection. To achieve bioinks with optimal viscosity and homogeneity for seamless extrusion and to achieve structural fidelity during 3D bioprinting, various mixing conditions were systematically optimized, focusing on key parameters such as stirring speed, temperature, and mixing duration. Firstly, a 1% (*w*/*v*) nanofibrillated cellulose (NFC) solution was prepared by premixing at 750 rpm and 23 °C for 24 h. This extended premixing significantly reduced aggregation, improving the solution’s compatibility compared to formulations without premixing.

Subsequently, NFC was incorporated into hybrid hydrogel formulations containing varying concentrations of alginate and carboxymethyl cellulose (CMC). Mixing followed a two-phase process: the first phase involved solely NFC at 750 rpm for 24 h, while the second phase included premixed NFC with alginate and CMC at 1150 rpm for 46 h, conducted at 23 °C. This dual-stage process achieved superior homogeneity and rheological stability.

Table 1 summarizes the conditions and outcomes of the various mixing steps for the preparation of optimum hybrid hydrogels used in custom bioink formulations.

To optimize turnover time and further enhance bioink properties, elevated temperatures were later integrated into the preparation process. Mixing at higher temperatures (90–130 °C) disrupted molecular interactions, facilitating the dissolution of rigid polymers while preserving NFC’s nanostructure. Elevated temperatures also reduced the energy barrier for molecular dispersion, preventing phase separation and particle aggregation. This approach significantly improved viscosity, blending, and stability, which are critical for extrusion-based bioprinting. For example, solutions containing CMC and alginate at 90 °C facilitated gelation, enhanced homogeneity, and maintained print fidelity. Prior research has demonstrated that temperature-controlled mixing is crucial for developing bioinks with enhanced performance characteristics, especially in polysaccharide-based formulations, underscoring the significance of these optimized conditions for 3D bioprinting applications [35,36,37].

In this study, a set of substrates was firstly developed for the formulation of bioinks aimed at examining the effect of extrusion pressure on the proliferation of *Chlorella* algae cells within various bioink substrates. Six custom bioinks (S1-N1, S1-C2, S1-A2:C1, S1-A2:N1, S1-A2, and S1-A1) are derived from this set of hydrogel substrates.

For the hydrogel formulation for bioink S1-N1, 1% NFC (*w*/*v*) at 65 °C was initially stirred for 45 min at 450 rpm with 30 mL of algae culture medium (ALGA-GRO, Carolina Biological Supply, Burlington, NC, USA), followed by an additional 40 min at 1000 rpm. For clarification purposes, the mixing temperatures indicated in this article for every mixed sample are temperature values of the heating surface of the magnetic stirrer at the start of the mixing process. In the case of bioink S1-C2, 2% CMC (*w*/*v*) at 90 °C was mixed with 30 mL of the algae culture medium for 30 min at 750 rpm, then continued for another 40 min at the same speed.

To prepare the hydrogel formulation for bioink S1-A2:C1, a mixture of 2% alginate and 1% CMC (*w*/*v*) at 130 °C was stirred with 30 mL of the algae culture medium for 15 min at 750 rpm, then continued for another 30 min at the same speed. For bioink S1-A2:N1, 2% alginate and 1% NFC (*w*/*v*) were mixed at 95 °C with 30 mL of the algae culture medium for 30 min at 750 rpm, followed by another 35 min at the same speed.

The hydrogel formulation for bioink S1-A2 was prepared by mixing 2% alginate (*w*/*v*) at 65 °C with 30 mL of the algae culture medium for 25 min at 1000 rpm, then continuing for an additional 90 min at the same speed. For bioink S1-A1, 1% alginate (*w*/*v*) at 65 °C was mixed with 30 mL of the algae culture medium for 15 min at 450 rpm, followed by an additional 85 min at 1000 rpm.

Table 2 summarizes the compositions and mixing conditions for hydrogel substrates aimed at formulating the S1 bioink series.

To replicate results from the S1 series and to develop custom bioink formulations aimed at investigating the impact of nanofibrillated cellulose (NFC) on cell proliferation, another set of hydrogel substrates was created. Custom bioinks derived from this second set of hydrogel substrates are: S2-A2 and S2-N1, S2-N1:A3, S2-N1:C0.5, S2-N1:C0.25, S2-A3:C0.5:N0.5, and S2-A3:C0.25:N0.5.

In the preparation of the hydrogel formulation for bioink S2-A2, 2% alginate (*w*/*v*) at 65 °C was mixed with 30 mL of the algae culture medium for 25 min at 1000 rpm, then continued for another 90 min at the same speed. For bioink S2-N1, 1% NFC (*w*/*v*) at 65 °C was stirred with 30 mL of the algae culture medium for 45 min at 450 rpm, followed by an additional 40 min at 1000 rpm.

For bioink S2-N1:A3, 1% NFC and 3% alginate (*w*/*v*) at 80 °C were mixed with 30 mL of the algae culture medium for 40 min at 450 rpm, then continued for another 2 h at 450 rpm and an additional 11 h at 1000 rpm. For bioink S2-N1:C0.5, 1% NFC and 0.5% CMC (*w*/*v*) at 90 °C were mixed with 30 mL of the algae culture medium for 40 min at 450 rpm, followed by an additional 2 h at 450 rpm and another 17 h at 1000 rpm. Similarly, the hydrogel formulation for bioink S2-N1:C0.25 was prepared by mixing 1% NFC and 0.25% CMC (*w*/*v*) at 90 °C with 30 mL of the algae culture medium for 40 min at 450 rpm, followed by an additional 2 h at 450 rpm and another 17 h at 1000 rpm.

For bioink S2-A3:C0.5:N0.5, a hydrogel formulation was derived with a mixture of 3% alginate, 0.5% CMC, and 0.5% NFC (*w*/*v*) at 90 °C stirred with 30 mL of the algae culture medium for 40 min at 450 rpm, then continued for another 2 h at 450 rpm and an additional 10 h at 1000 rpm. Finally, for bioink S2-A3:C0.25:N0.5 a hydrogel formulation was prepared by mixing 3% alginate, 0.25% CMC, and 0.5% NFC (*w*/*v*) at 90 °C with 30 mL of the algae culture medium for 40 min at 450 rpm, followed by an additional 2 h at 450 rpm and another 10 h at 1000 rpm.

Table 3 summarizes the compositions and mixing conditions for hydrogel substrates aimed at formulating the S2 bioink series.

### 2.3. Cell Preparation and Culture

*Chlorella* cells were cultured in vitro in an algal medium under controlled conditions to optimize cell growth, ensuring appropriate nutrient concentrations and continuous aeration for cellular respiration and metabolism. Exposure to sunlight during proliferation stimulated photosynthesis, a critical metabolic process enhancing their activity and promoting vitality. This sunlight exposure replicated natural environmental conditions, preparing cells for subsequent applications. After achieving the desired growth, cells were carefully harvested and isolated via centrifugation while maintaining integrity and viability. Subsequently, cells were introduced into hydrogel substrates to create bioinks, with the hydrogel providing mechanical stability and spatial organization during the bioprinting process.

### 2.4. Preparation of Bioinks

To initiate the initial algae cell suspension (Set 1), 0.1 mL of harvested *Chlorella* algae sourced from Carolina Biological Supply, Burlington, NC, USA, was aliquoted into a 10 mL deionized water in a 15 mL centrifuge tube obtained from Fisher Scientific Inc., Pittsburgh, PA, USA. This process yielded a cell count of 51.5 × 10^6^ cells/mL, determined using a hemocytometer from Fristaden Lab, Reno, NV, USA, and a trinocular LED light microscope (M837, OMAX Microscope, Sacramento, CA, USA). For the preparation of another algae cell suspension (Set 2), 0.1 mL of harvested *Chlorella* algae was aliquoted into a 10 mL deionized water in a 15 mL tube, resulting in a cell count of 55.3 × 10^6^ cells/mL. Before cell count estimation, the hemocytometer was cleaned with 70% ethanol and dried. The cell suspension was thoroughly mixed, and a small volume was loaded into the hemocytometer chamber for cell settling. Cells within defined grids were counted using a microscope, and concentrations were calculated based on grid size and dilution factor to obtain accurate cell counts.

An initial set of bioinks was formulated to investigate the impact of extrusion pressure on the post-printing proliferation of *Chlorella* algae cells within each bioink substrate. Bioinks were respectively prepared as S1-N1, S1-C2, S1-A2:C1, S1-A2:N1, S1-A2, and S1-A1 by seeding 0.15 mL of Set 1 cell suspension (51.5 × 10^6^ cells/mL) onto 10 mL of substrates N1, C2, A2:C1, A2:N1, A2, and A1 in a 15 mL tube. For control experiments, 0.15 mL of Set 1 cell suspension was also seeded onto 10 mL of ALGA-GRO media.

To replicate the results obtained with bioinks S1-A2 and S1-N1, two samples in a second set of bioinks (S2-A2 and S2-N1) were created. This involved seeding 0.3 mL of Set 2 cell suspension (55.3 × 10^6^ cells/mL) onto 15 mL of substrates A2 and N1. Control experiments in this replication process included seeding 0.3 mL of Set 2 cell suspension onto 15 mL of ALGA-GRO media.

To investigate the effect of nanofibrillated cellulose (NFC) presence in substrates on *Chlorella* algae cell proliferation within microalgae-based bioinks, five additional samples were created in the second set of bioinks. These samples were prepared sequentially by seeding 0.3 mL of Set 2 cell suspension (53.3 × 10^6^ cells/mL) onto 15 mL of substrates N1:C0.5, N1:C0.25, N1:A3, A3:C0.5:N0.5, and A3:C0.25:N0.5 to obtain bioinks S2-N1:C0.5, S2-N1:C0.25, S2-N1:A3, S2-A3:C0.5:N0.5, and S2-A3:C0.25:N0.5, respectively.

**Figure 3:**
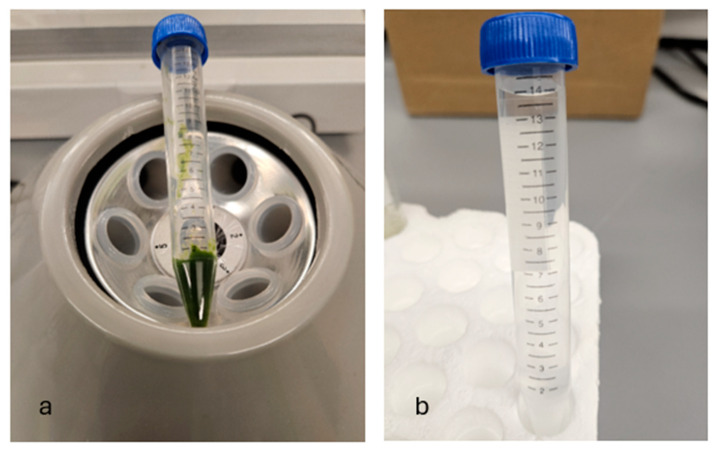
Centrifuged cell suspension (**a**), and hydrogel substrate in tube before seeding with cell suspension (**b**).

**Figure 4:**
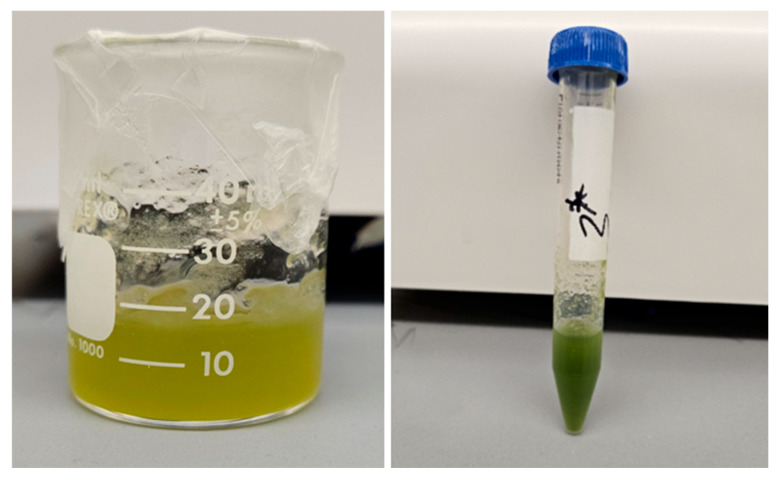
Bioinks made up of hydrogel substrates and cell suspensions.

Images from laboratory sessions during this study demonstrating the distinction between a cell suspension, hydrogel substrate, and bioink are presented in Figure 3a,b and Figure 4. Table 4 and Table 5 also present the nomenclature of the hydrogel substrates and their corresponding bioink formulations.

### 2.5. Estimation of Cell Growth Rate in Substrates

Bioink samples were prepared by adding microalgae cell suspension to hydrogel substrates, and cuvettes were filled with 3.7 mL of these samples (ON 67.755, Sarstedt AG & CO., Numbrecht, Germany).

Considering the green color of *Chlorella* microalgae and based on literature [38], which indicates that the wavelength range for visible green light typically falls between 495 nm and 570 nm, a spectrophotometer (721 Visible, Ningbo Justop Medical Instruments, Ningbo, China), set to wavelengths ranging from 450 nm to 600 nm, was used to carry out preliminary absorbance measurements for the bioink samples. Among these wavelengths, 450 nm was identified as the most suitable.

**Figure 5:**
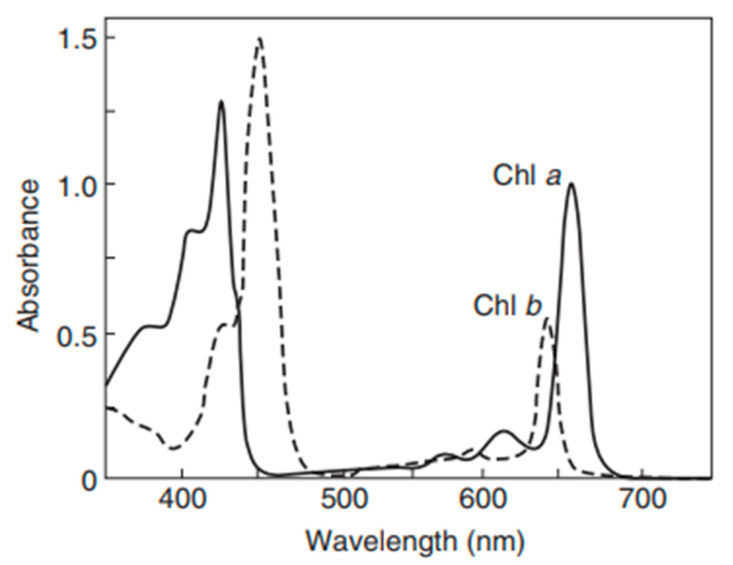
Absorption spectra of freshly isolated Chl a and Chl b in diethyl ether [40].

Studies have also shown that light-harvesting chlorophylls a and b exhibit absorption peaks (depicted in Figure 5) between 429 nm and 472 nm, as well as between 642 nm and 674 nm, providing further insight into the absorption characteristics of these pigments [39]. To confirm these findings, the custom bioink formulations used in this study were analyzed through spectrophotometric measurements at wavelengths of 450 nm, 500 nm, 550 nm, 600 nm, and 650 nm. The optimal wavelengths for *Chlorella* microalgae absorbance were found to be near 450 nm and 650 nm, which aligns with previous research on the absorption properties of chlorophyll. Between 500 nm and 600 nm, absorbance readings were predominantly negative, supporting the conclusion that 450 nm and 650 nm are the most reliable wavelengths for absorbance measurements across bioink samples in this study. These results validate both wavelengths as effective for evaluating *Chlorella* cell growth density.

Spectrophotometer calibration involves blank solutions with matching refractive properties and setting a benchmark absorbance of zero using an empty, regular transparent cuvette. Calibration involves setting the parameters for absorbance measurement at a predetermined wavelength. Upon insertion of bioink samples contained in cuvettes into the spectrophotometer, measurements of absorbance corresponding to the light absorbed by the bioink at the set wavelength were recorded for subsequent analysis. The residual absorbance values were computed by subtracting the absorbance values of the suspension/substrate mixture from those of the respective hydrogel substrate.

Samples were maintained under sterile conditions in a chemical fume hood (Labanco Corporation, Kansas City, MO, USA), illuminated by Keystone Direct Drive 2011j LED tubes (Keystone Technologies, Lansdale, PA, USA).

**Figure 6:**
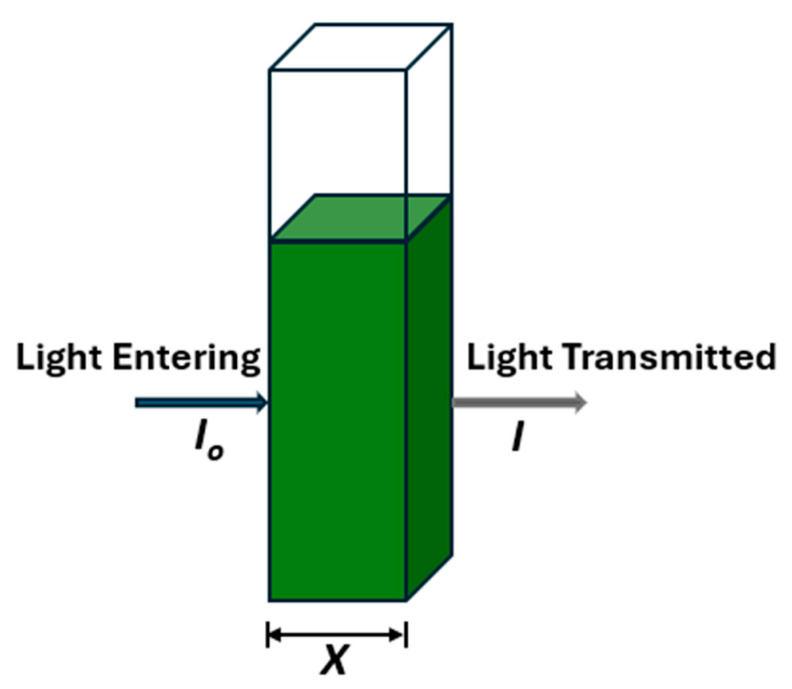
Schematic of light transmission through a cuvette.

The Beer-Lambert law is leveraged in this study to link absorbance to cell density. The law assumes homogeneity in the distribution of the absorbing substance across the sample, inherently accommodating concentration variations. Also, its linear relationship ensures absorbance stays directly proportional to concentration as long as changes occur uniformly with path length [41]. When a sample of a microalgae-based bioink is confined within a container (like a cuvette) with a path-length X (Figure 6), having walls that are parallel, planar, and non-absorbing at the wavelength of interest, the relationship between the incident light and the transmitted light is established using Beer–Lambert’s law, traditionally stated as in Equations (1a) and (1b)

(1a) $$Abs\equiv \ln\left\{\frac{I_{0}}{I}\right\}=\varepsilon cX$$

(1b) $$Abs\equiv \frac{\varepsilon n}{S}=\varepsilon Xc_{average}$$

where *Abs* is the absorbance, $I_{0}$ is the intensity of monochromatic light entering the solution perpendicularly to one face, and *I* is the intensity of light exiting the solution through the opposite face. The constant *ε* is known as molar absorptivity with an SI unit of m^2^ mol^−1^. *S* is the cross-sectional area of the cell and *n* is the number of moles of absorber present, and c is the concentration.

The transmittance T, which can be expressed with Equation (2):

(2) $$T\equiv \left\{\frac{I}{I_{0}}\right\}=\exp\left\{-\varepsilon cX\right\}$$

gives its definition and its interpretation according to Beer–Lambert’s law. Equation (3) shows the differential form of Beer–Lambert’s law, which was integrated to obtain Equations (1a) and (1b) above.

(3) $$\frac{dI}{dX}=-\varepsilon cI$$

where *X* is the dimension along which the light travels. This differential form of the equation is fundamental, and it is not anchored to any geometry or experiment.

### 2.6. Rheological Properties of Hybrid Substrates

To investigate the effect of extrusion pressure on post-fabrication variations in cell density and morphology, rheological measurements were conducted using a rotational rheometer (ARES-LS2, TA Instruments, New Castle, DE, USA) equipped with parallel plate geometry (20 mm flat plate). The viscosity and shear stress values of the hydrogel substrates were determined by varying the shear strain rate from 1.0 s^−1^ to 100 s^−1^, with a 0.5 mm gap width set for each measurement at 25 °C. The flow characteristic parameters of each hydrogel substrate can be obtained using the Power Law equation (Equation (4)) [42]:

(4) $$\eta =K{\dot{\gamma }}^{N-1}$$

where *η* represents viscosity, $\dot{\gamma }$ stands for shear rate, and *K* and *N* denote the shear thinning coefficients.

For a Newtonian fluid, the relationship between viscosity and shear stress can be determined by Equation (5).

(5) $$\tau (y)=\eta \frac{𝜕u}{𝜕y}$$

where *τ*(*y*) represents shear stress, u is the flow velocity along the boundary, and y denotes the height above the boundary.

### 2.7. Printability Test on Hydrogel and Bioink Formulations

**Figure 7:**
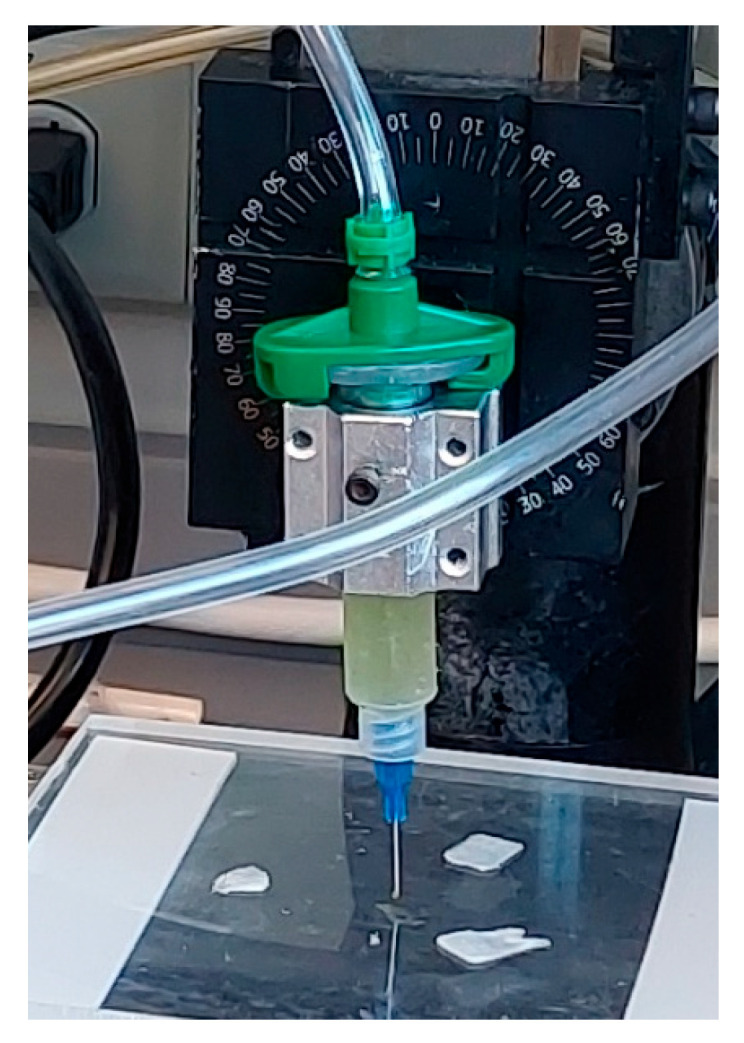
Dispense assembly of custom bioprinter at UMaine Digital and Additive Manufacturing Lab.

**Figure 8:**
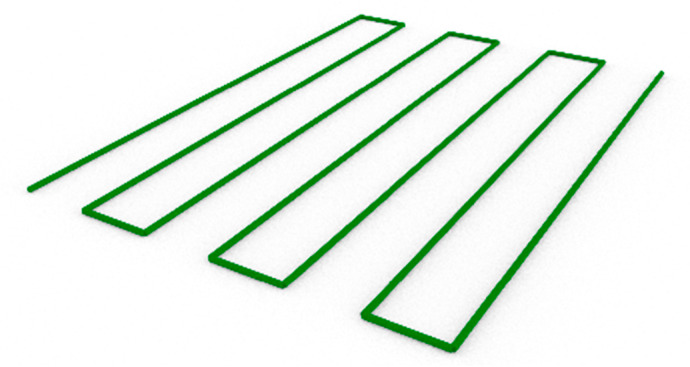
3D model for experimental bioprinting construct.

In this study, a custom XYZ-axis micro-extrusion 3D bioprinter developed for biomedical projects at our Additive and Digital Manufacturing Laboratory is used to fabricate experimental constructs with microalgae-laden bioinks under sterile conditions. The dispensing assembly (Figure 7) features a disposable syringe barrel (EFD, Nordson, Westlake, OH, USA) and dispensing tip (EFD, Nordson, Westlake, OH, USA) with a 410 μm inner diameter for precise dispensing onto a stationary print bed. Visual Basic scripting was used to generate G-Codes to create vectorized toolpaths, ensuring accurate bioink deposition. Toolpath simulation aids in optimizing printing by visualizing the printing nozzle trajectory, facilitating the assessment of printability for various applications. This custom bioprinter’s development emphasizes tailored functionality for biomedical research, offering precision and versatility. Utilizing industry-standard components, like the EFD syringe barrel and dispensing tip, ensures reliability and compatibility with established bioprinting protocols and practices [43]. A 3D model for the experimental bioprinting construct used in this study is given in Figure 8.

## 3. Results and Discussion

The findings presented in this study underscore the critical importance of rheological performance of specific hydrogels as an identifier of printability, and printing parameters to enhance the efficacy of bioprinted constructs. Through our comprehensive investigation into the use of absorbance of specific bioinks as indication of the suitability for *Chlorella* microalgae cell proliferation under 3D bioprinting conditions. We have gained valuable insights into the distinct and synergistic effects of various hydrogel components on cell behavior and bioink performance. These results are interpreted to provide insight into future paths for high-performance bioinks for plant cells printing. 

### 3.1. Spectrophotometric and Rheological Analysis

**Figure 9:**
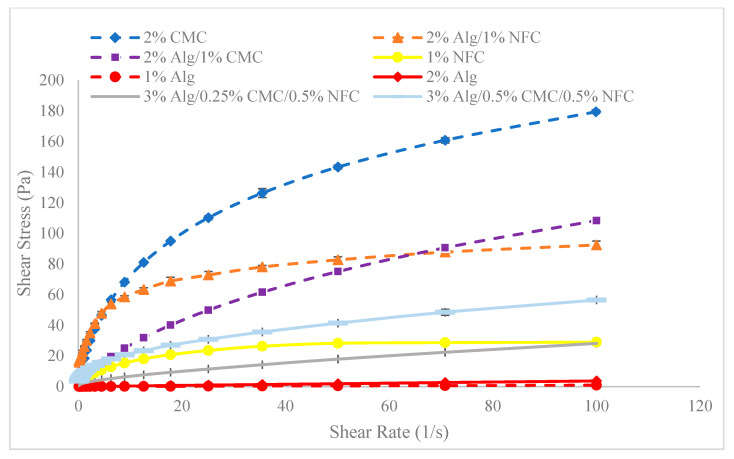
Comparison of rheological characteristics of hydrogel formulations prepared to serve as substrates for custom bioinks.

Hydrogels serve as the base of a bioink, being the substrate to which plant cells are added to form a bioink. The analysis of the rheological characteristics of hydrogel formulations, as depicted in Figure 9, provides insights into their suitability, with desired viscoelastic properties which will allow flexible printing while maintaining post-fabrication shape and cell viability. 

**Figure 10:**
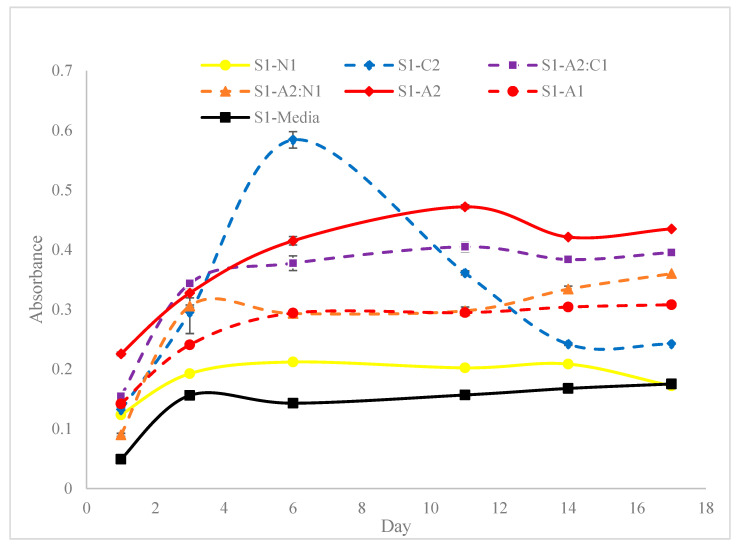
Relative variation of absorbance for the first set of bioinks at a wavelength of 450 nm and a cell count of $51.5\times 10^{6}\;cells/mL$.

**Figure 11:**
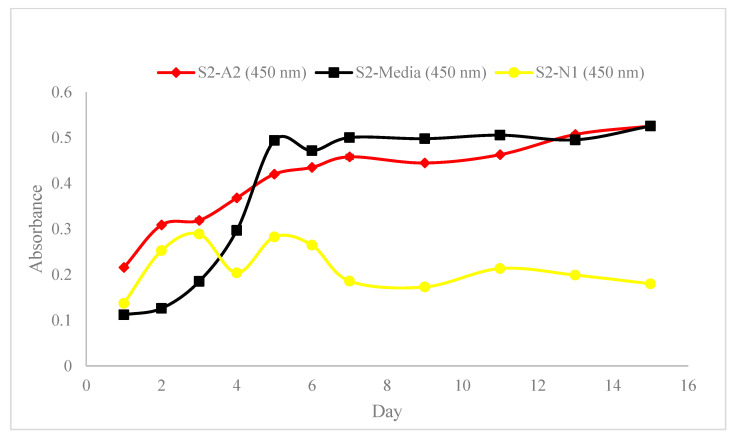
Replication of absorbance variation in ‘Bioinks S1-A2, S1-Media, and S1-N1’ with ‘Bioinks S2-A2, S2-Media, and S2-N1’, respectively, at a wavelength of 450 nm and a cell count of $55.3\times 10^{6}\;cells/mL$.

**Figure 12:**
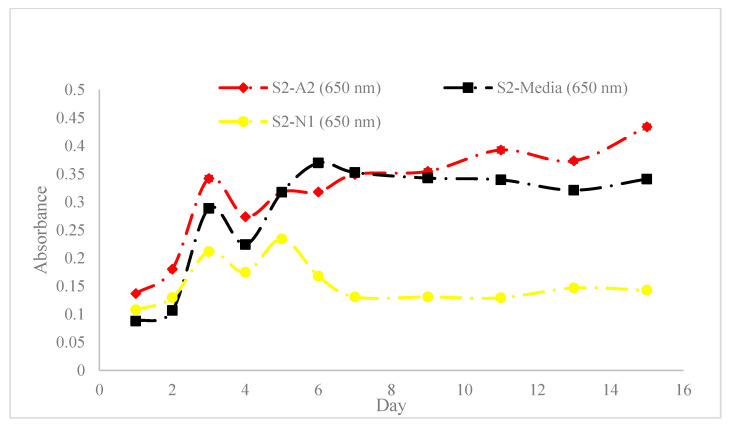
Replication of absorbance variation in ‘Bioinks S1-A2, S1-Media and S1-N1’ with ‘Bioinks S2-A2, S2-Media, and S2-N1’, respectively, at a wavelength of 650 nm and a cell count of $55.3\times 10^{6}\;cells/mL$.

Figure 10, Figure 11 and Figure 12 show the absorbance variations at different wavelengths (450 nm and 650 nm) and cell concentrations, highlighting the reproducibility and consistency of the bioinks. Specifically, the relative variation in absorbance for the first set of bioinks (Figure 10) and its replication across different formulations (Figure 11 and Figure 12) indicate robust formulation protocols that ensure reliable performance across different batches.

**Figure 13:**
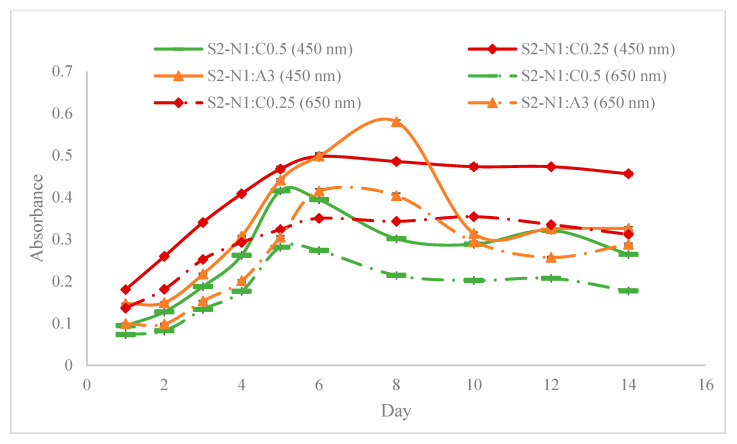
Representation of the effect of NFC content in bioink substrates on cell density variation.

Figure 13 reveals that higher NFC concentrations can potentially hinder light penetration, thereby affecting the growth of chlorophyll-based cells. This finding aligns with our hypothesis and suggests that careful optimization of NFC content is critical to balancing adhesive characteristics and optical properties.

**Figure 14:**
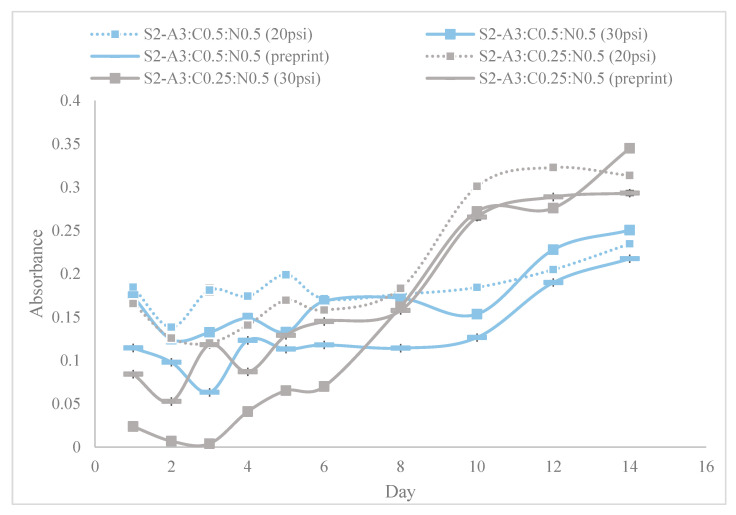
Cell density variation in microalgae constructs derived from tri-hydrogel substrate, bio-printed at different printing pressures.

Figure 14 provides a detailed examination of cell density variations in microalgae constructs derived from tri-hydrogel substrates under different printing pressures. The results demonstrate that lower printing pressures yield better cell viability shortly after the bioprinting process, likely due to reduced shear stress during the extrusion process. Interestingly, cell multiplication appears not to be negatively impacted during the post-bioprinting culture of the microalgae constructs.

**Figure 15:**
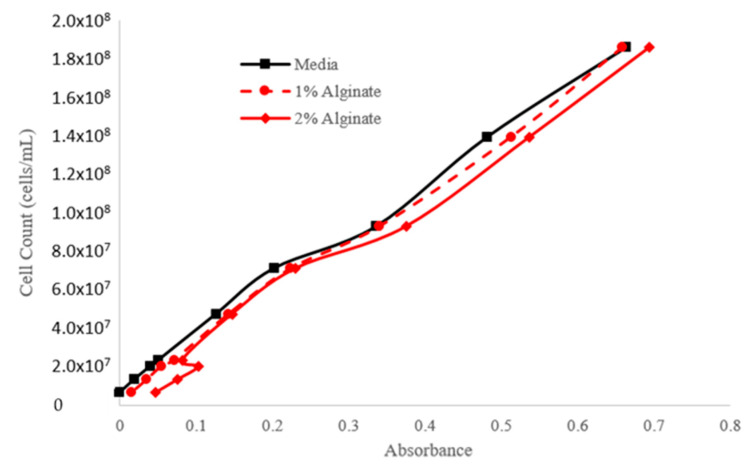
Cell Count–Absorbance calibration curve (absorbance recorded at a wavelength of 450 nm).

**Figure 16:**
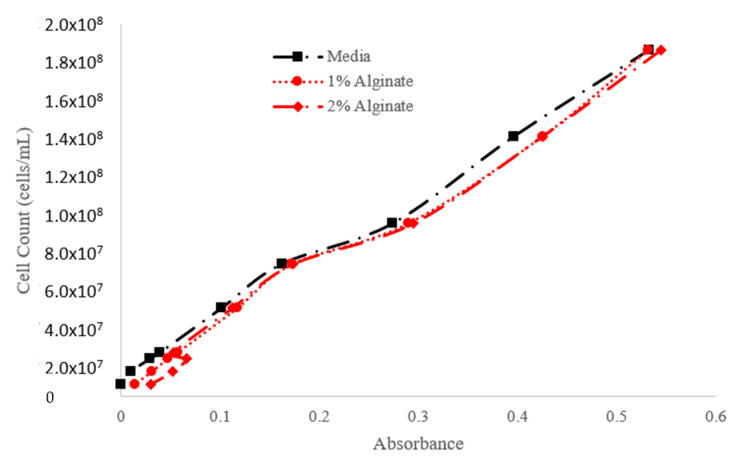
Cell Count–Absorbance calibration curve (absorbance recorded at a wavelength of 650 nm).

The calibration curves presented in Figure 15 and Figure 16 establish a strong correlation between cell count and absorbance at 450 nm and 650 nm, respectively. These curves are essential for accurately estimating cell density in experimental setups, facilitating precise monitoring of cell growth and proliferation.

### 3.2. Comparative Visual Assessment of Experimental Constructs

**Figure 17:**
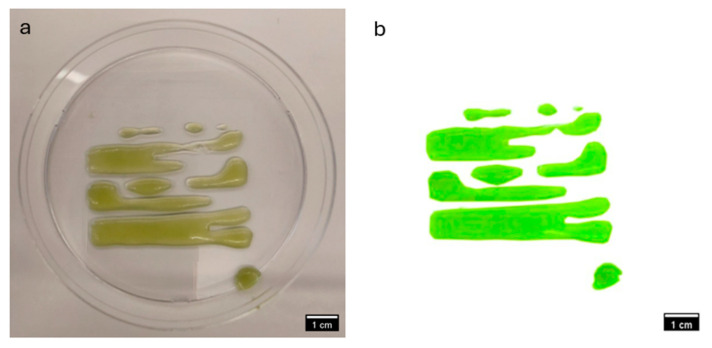
(**a**,**b**). Image of the experimental construct from Bioink SE-N1 (bioprinted from experimental bioink derived from 1% NFC *w*/*v*).

**Figure 18:**
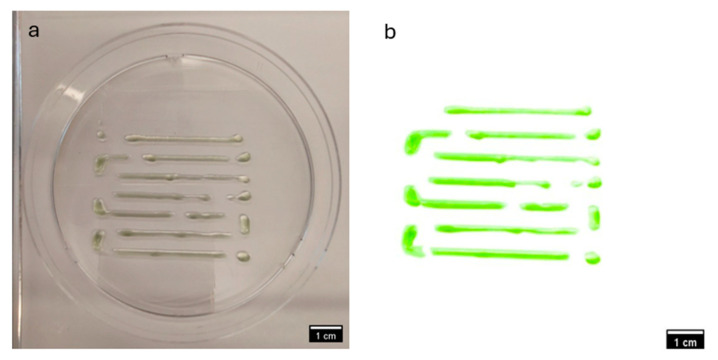
(**a**,**b**). Image of the experimental construct from Bioink SE-C2 (bioprinted from experimental bioink derived from 2% CMC *w*/*v*).

The visual assessment of experimental constructs is depicted in Figure 17 and Figure 18 provides valuable insights into the comparative performance off CMC-based and NFC-based experimental bioinks. Figure 17 showcases constructs derived from Bioink SE-N1, formulated with 1% NFC (*w*/*v*), while Figure 18 displays constructs from Bioink SE-C2, formulated with 2% CMC (*w*/*v*).

Upon visual inspection, it is evident that the constructs from Bioink SE-C2 exhibit superior structural integrity and overall quality compared to those from Bioink SE-N1. The constructs from Bioink SE-C2 appear to have a more uniform and well-defined morphology, with smoother surfaces and better consolidation of material layers. This observation suggests that the CMC-based experimental bioink yields better constructs in terms of printability and structural fidelity.

The enhanced performance of the CMC-based experimental bioink may be attributed to several factors, including the superior shear-thinning properties and stability of CMC, as well as its biocompatibility and hydrophilic nature. These properties likely contribute to improved extrusion behavior and material deposition during the printing process, resulting in more robust and well-defined constructs.

In contrast, the constructs from Bioink SE-N1, derived from NFC-based experimental bioink, appear to exhibit inconsistencies in layer deposition and structural integrity. This may be indicative of challenges associated with the rheological properties and printability of NFC-based bioinks, such as poor shear-thinning behavior or inadequate material adhesion.

Overall, the visual comparison of experimental constructs supports the notion that CMC-based experimental bioinks outperform NFC-based bioinks in terms of construct quality and printability.

Table 6 summarizes the physical properties of experimental bioinks and process variables for bioprinting, providing a comprehensive overview of the experimental parameters. The consistency in extrusion pressure and the detailed measurement of bioink mass and volume underscore the rigorous control of experimental conditions. Notably, the significant percentage difference in the volume of constructs between Bioink SE-N1 and Bioink SE-C2 highlights the variability in bioink performance, which can be attributed to differences in formulation and handling.

### 3.3. Insights into Bioink Formulation for Improved Bioprinting Performance

Findings presented in this section underscore the importance of optimizing bioink formulations and printing parameters to enhance the efficacy of bioprinted constructs. The data suggest that CMC-based bioinks, due to their stable viscosity and superior shear-thinning properties, are more suitable for applications requiring consistent performance and reliable cell growth, especially for chlorophyll-based cells. Future studies should focus on further refining these formulations and exploring the synergistic effects of combining different hydrogel components to achieve the desired balance between mechanical strength, transparency, and cell viability.

Based on the experimental findings, it is evident that Carboxymethyl cellulose (CMC) is more suitable than Nanofibrillated Cellulose (NFC) for green bioinks, particularly for applications involving chlorophyll-based cells like algae. CMC’s superior shear-thinning properties, transparency, and biocompatibility appear to make it an ideal candidate for bioinks that support both cell growth and photosynthesis while maintaining high printability and structural fidelity. CMC’s hydrophilic nature and enhanced nutrient diffusion capabilities could have further contributed to its suitability for sustaining cell viability and growth in printed constructs. These properties ensure that CMC-based bioinks provide a conducive environment for cells, promoting proliferation, especially in the first few days after the initiation of the culturing process.

The stability of CMC’s viscosity over time, coupled with its favorable rheological properties, underscores its reliability in 3D printing applications. This stability is important for ensuring consistent performance and reproducibility in biofabrication processes. By optimizing CMC concentration and incorporating suitable reinforcing agents or crosslinking methods, it is possible to enhance the mechanical properties of CMC-based bioinks while preserving their printability and biocompatibility. Such strategies ensure that CMC-based bioinks can deliver consistent, reliable performance, making them well-suited for tissue engineering and regenerative medicine applications involving photosynthetic cells.

Using color variation as a proxy for assessing cell viability and proliferation in chlorophyll-based plant cells within CMC-based bioinks is a practical and effective approach. Visual inspection, digital imaging, and spectrophotometric measurements allow for monitoring cell health and growth over time. This method is particularly valuable in scenarios where direct viability assays are challenging to implement. The consistent viscosity and favorable properties of CMC-based bioinks further support their use in such applications, ensuring reliable and reproducible results.

The study also highlighted the significant role of alginate as a potent substrate in fostering accelerated cell density and concentration. Alginate appeared to facilitate cell proliferation and enhance morphology, and this characteristic highlights its importance as a functional component of bioink. However, the inherent low viscosity of alginate-only bioinks renders them unsuitable for standalone 3D bioprinting applications, emphasizing the necessity of combining alginate with other hydrogel components to achieve the desired viscosity and mechanical properties. Hybrid substrates combining alginate and CMC demonstrated superior performance, effectively addressing the limitations of CMC-only bioinks and enhancing overall cell growth and bioink stability.

The use of bioinks in 3D bioprinting, particularly with photosynthetic organisms like algae, presents significant potential for advancing sustainable practices such as carbon capture and bioremediation. By creating customized structures tailored for microbial activity, bioinks can enhance environmental remediation efforts by providing increased surface area for microorganisms, thereby improving their efficiency in breaking down pollutants. These bioinks support sustainable bioprinting by integrating photosynthetic organisms, like algae, which can be employed in eco-friendly applications such as capturing carbon from the atmosphere or degrading environmental contaminants. This approach enables the development of tailored materials that not only contribute to pollution reduction but also support the creation of self-sustaining, bioactive systems capable of addressing complex environmental challenges.

## 4. Conclusions

Our study provides a comprehensive exploration of hydrogel formulations and their impact on cell proliferation, offering critical insights for advancing bioprinting techniques. Through detailed rheological analyses (Figure 9), we demonstrate the importance of optimizing viscoelastic properties to enhance both printability and cell viability, which is crucial for effective biofabrication. As demonstrated in Figure 10, Figure 11 and Figure 12, bioinks produced in this study are consistent and reproducible due to robust formulation protocols. Additionally, error bars in these figures confirm the precision of the measurements, showing the variability in our data and supporting the reliability of our findings. Our findings regarding NFC content (Figure 13) highlight the balance needed to support cell growth, particularly in chlorophyll-based cells like algae.

Examining cell density variations in microalgae constructs (Figure 14) demonstrates the role of printing pressures in ensuring optimal post-bioprinting cell viability. Additionally, establishing strong correlations between cell count and absorbance (Figure 15 and Figure 16) provides essential tools and a rapid method for accurate cell density estimation and monitoring. Importantly, our findings suggest the preferential use of CMC-based bioinks due to their stable viscosity, superior shear-thinning properties, and biocompatibility, making them ideal for supporting cell proliferation while maintaining high printability and structural fidelity. The practical use of color variation as a proxy for assessing cell viability within CMC-based bioinks offers a valuable approach in challenging scenarios for direct viability assays.

Alginate can play an important role in a substrate by enhancing cell density and morphology. While alginate-only bioinks have limitations in standalone 3D bioprinting due to low viscosity, hybrid formulations combining alginate with CMC show enhanced bioink stability and overall cell growth. By investigating the effects of different hydrogel materials—alginate, Nano-fibrillated Cellulose (NFC), and CarboxyMethyl Cellulose (CMC)—on cell proliferation, growth, and morphology, we found that alginate-based bioinks supported higher cell density and increased cell proliferation, while NFC exhibited reduced shear thinning compared to CMC.

We recommend that future research explore a broader range of extrusion pressures to assess their direct effects on cell morphology and overall print quality. Investigating how different pressures influence cell shape and viability could provide valuable insights into improving construct reproducibility and enhancing the quality of printed tissue-engineered constructs. This approach will help refine the bioprinting process and improve the preservation of cell integrity, a critical factor for advancing regenerative medicine. Additionally, future work should focus on refining the ratio of CMC scaffolding with bi- or tri-hydrogel substrates, prioritizing printability before enhancing cell growth. Comparing bioink formulations at varying concentrations and under different environmental conditions will also provide a clearer understanding of their effects on cell proliferation and bioink performance. These combined efforts will contribute to advancing bioprinting techniques, ultimately improving the quality of tissue-engineered constructs and enhancing the preservation of cell integrity during printing.

## Figures and Tables

**Table 1:** Summary of Mixing Conditions and Outcomes for Hybrid Hydrogel Preparation.

Step	Condition	Time	Speed (rpm)	Temperature (°C)	Outcome
Premixing NFC solution	1% (*w*/*v*) NFC, under controlled conditions	24 h	750	23	Reduced aggregation
Two-phase mixing	Hybrid hydrogel formulation with NFC, alginate, CMC	Phase 1: 24 h, Phase 2: 46 h	Phase 1: 750, Phase 2: 1150	23	Superior homogeneity

**Table 2:** Hydrogel Substrate Preparation for S1 Bioink Series.

Target Bioink	Hydrogel Composition (% *w*/*v*)	Temperature (°C)	Mixing Duration	Mixing Speeds (rpm)
S1-N1	1% NFC	65	0.75 h + 0.67 h	450 → 1000
S1-C2	2% CMC	90	0.5 h + 0.67 h	750
S1-A2:C1	2% Alginate, 1% CMC	130	0.25 h + 0.5 h	750
S1-A2:N1	2% Alginate, 1% NFC	95	0.5 h + 0.58 h	750
S1-A2	2% Alginate	65	0.42 h + 1.5 h	1000
S1-A1	1% Alginate	65	0.25 h + 1.42 h	450 → 1000

**Table 3:** Hydrogel Substrate Preparation for S2 Bioink Series.

Target Bioink	Hydrogel Composition (% *w*/*v*)	Temperature (°C)	Mixing Duration	Mixing Speeds (rpm)
S2-A2	2% Alginate	65	0.42 h + 1.5 h	1000
S2-N1	1% NFC	65	0.75 h + 0.67 h	450 → 1000
S2-N1:A3	1% NFC, 3% Alginate	80	0.67 h + 2 h + 11 h	450 → 1000
S2-N1:C0.5	1% NFC, 0.5% CMC	90	0.67 h + 2 h + 17 h	450 → 1000
S2-N1:C0.25	1% NFC, 0.25% CMC	90	0.67 h + 2 h + 17 h	450 → 1000
S2-A3:C0.5:N0.5	3% Alginate, 0.5% CMC, 0.5% NFC	90	0.67 h + 2 h + 10 h	450 → 1000
S2-A3:C0.25:N0.5	3% Alginate, 0.25% CMC, 0.5% NFC	90	0.67 h + 2 h + 10 h	450 → 1000

**Table 4:** First set of bioinks (from cell suspension with $51.5\times 10^{6}\;cells/mL$).

Bioink	Hydrogel Substrate Composition	Nomenclature for Substrate
S1-N1	1% NFC	N1
S1-C2	2% CMC	C2
S1-A2:C1	2% Alginate/1% CMC	A2:C1
S1-A2:N1	2% Alginate/1% NFC	A2:N1
S1-A2	2% Alginate	A2
S1-A1	1% Alginate	A1
S1-Media	In Media Only	Media

**Table 5:** Second set of bioinks (from cell suspension with $53.3\times 10^{6}\;cells/mL$).

Bioink	Hydrogel Substrate Composition	Nomenclature for Substrate
S2-A2	2% Alginate	A2
S2-Media	In Media Only	Media
S2-N1	1% NFC	N1
S2-N1:C0.5	1% NFC/0.5% CMC	N1:C0.5
S2-N1:C0.25	1% NFC/0.25% CMC	N1:C0.25
S2-N1:A3	1% NFC/3% Alginate	N1:A3
S2-A3:C0.5:N0.5	3% Alginate/0.5% CMC/0.5% NFC	A3:C0.5:N0.5
S2-A3:C0.25:N0.5	3% Alginate/0.25% CMC/0.5% NFC	A3:C0.25:N0.5

**Table 6:** Physical properties of experimental bioinks and process variables for experimental bioprinting.

	Bioink SE-N1	Bioink SE-C2
Extrusion pressure (psi)	15	15
Mass of empty tube (g)	6.491	6.544
Mass of loaded bioink tube (g)	17.275	17.841
Net mass of bioink in tube (g)	10.784	11.297
Volume of bioink in tube (mL)	11	11.5
Density of bioink (g/mL)	0.980364	0.982348
Mass of empty petri dish (g)	8.091	8.013
Mass of petri dish with construct (g)	9.559	8.213
Net mass of construct (g)	1.468	0.2
Actual volume of construct (mL)	1.497404	0.203594
Theoretical volume of construct (mL)	0.08	0.08
Percentage difference in volume of construct (%)	1871.754	254.4923

## Data Availability

The raw data supporting the conclusions of this article will be made available by the authors on request due to privacy reason.
